# NOD2/RIG-I Activating *Inarigivir* Adjuvant Enhances the Efficacy of BCG Vaccine Against Tuberculosis in Mice

**DOI:** 10.3389/fimmu.2020.592333

**Published:** 2020-12-07

**Authors:** Arshad Khan, Vipul K. Singh, Abhishek Mishra, Emily Soudani, Pearl Bakhru, Christopher R. Singh, Dekai Zhang, David H. Canaday, Anjaneyulu Sheri, Seetharamaiyer Padmanabhan, Sreerupa Challa, Radhakrishnan P. Iyer, Chinnaswamy Jagannath

**Affiliations:** ^1^ Department of Pathology and Genomic Medicine, Center for Human Infectious Diseases, Houston Methodist Research Institute, Weill Cornell Medicine, Houston, TX, United States; ^2^ Institute of Biosciences and Technology, Texas A&M Health Science Center, Houston, TX, United States; ^3^ Division of Infectious Diseases and HIV Medicine, Case Western Reserve University, Cleveland, OH, United States; ^4^ Spring Bank Pharmaceuticals, Inc., Hopkinton, MA, United States

**Keywords:** tuberculosis, BCG vaccine, inarigivir, adjuvants, NOD2, RIG-I, antigen presentation, inflammasome

## Abstract

Tuberculosis (TB) caused by *Mycobacterium tuberculosis* (MTB) kills about 1.5 million people each year and the widely used Bacille Calmette-Guérin (BCG) vaccine provides a partial protection against TB in children and adults. Because BCG vaccine evades lysosomal fusion in antigen presenting cells (APCs), leading to an inefficient production of peptides and antigen presentation required to activate CD4 T cells, we sought to boost its efficacy using novel agonists of RIG-I and NOD2 as adjuvants. We recently reported that the dinucleotide SB 9200 (Inarigivir) derived from our small molecule nucleic acid hybrid (SMNH)^®^ platform, activated RIG-I and NOD2 receptors and exhibited a broad-spectrum antiviral activity against hepatitis B and C, Norovirus, RSV, influenza and parainfluenza. Inarigivir increased the ability of BCG-infected mouse APCs to secrete elevated levels of IL-12, TNF-α, and IFN-β, and Caspase-1 dependent IL-1β cytokine. Inarigivir also increased the ability of macrophages to kill MTB in a Caspase-1-, and autophagy-dependent manner. Furthermore, Inarigivir led to a Capsase-1 and NOD2- dependent increase in the ability of BCG-infected APCs to present an Ag85B-p25 epitope to CD4 T cells *in vitro*. Consistent with an increase in immunogenicity of adjuvant treated APCs, the Inarigivir-BCG vaccine combination induced robust protection against tuberculosis in a mouse model of MTB infection, decreasing the lung burden of MTB by 1-log10 more than that afforded by BCG vaccine alone. The Inarigivir-BCG combination was also more efficacious than a muramyl-dipeptide-BCG vaccine combination against tuberculosis in mice, generating better memory T cell responses supporting its novel adjuvant potential for the BCG vaccine.

## Introduction

*Mycobacterium tuberculosis* (MTB) kills more than 1.5 million people each year and is the leading cause of death due to infections (1). Despite the high infectivity rate of the pathogen, 90% of people do not develop active disease and instead show a latent infection lasting decades (LTBI) (2). Although anti-tuberculosis immunity is multifactorial involving many types of innate and adaptive immune cells, Th1 immunity mediated by macrophages (MΦs), dendritic cells (DCs) (Antigen Presenting Cells; APCs), CD4, CD8 T cells and non-canonical T cells appears to be the major defense mechanism (3). Paradoxically, MΦs are also the reservoirs of MTB, and require either T cell derived IFN-γ mediated activation to kill MTB through nitric oxide or lyse MTB infected MΦs through perforin-granulysin from cytotoxic CD8 T cells (4).

BCG, an attenuated strain of *Mycobacterium bovis* has been used as a vaccine against tuberculosis since 1921, and nearly a billion doses have been given to infants so far with remarkably fewer side effects. Although BCG is known to effectively protect infants against disseminated forms of TB, it provides a variable (0–80%) protection against pulmonary tuberculosis and appears to be ineffective in protecting against adult tuberculosis (5, 6). The poor efficacy of BCG vaccine has been suggested to be due to 1) the lack of the RD1 region encoding for many immunodominant antigens, 2) vaccine strain-dependent variation, 3) exposure of infants to parasitic infestations which may skew Th1 to Th2 responses, and 4) heterogeneity of population genetics (5–8). Paradoxically, BCG is also known to induce “trained immunity” which protects against unrelated bacterial and viral infections of children and even against an experimental yellow fever virus infection of adults (9, 10). It is relevant to note here that, countries where primary BCG immunization is practiced report significantly less deaths due to COVID-19 (11). Therefore, BCG vaccine is now being repurposed for protection against SARS-CoV-2 infections (12, 13).

Although BCG is routinely used in many countries, an often-overlooked issue is the observation that the neonatal immune system is immature and antigen presenting cells (APCs) of neonates require adjuvant mediated stimulation to elicit robust cytokine responses to antigens (14). It has also been suggested that premature neonatal immune cells may not induce strong enough Th1 immune responses that can last for longer period of time (15). Poor processing and presentation of antigens generated from BCG at the site of vaccination may also negatively impact the T cell proliferation and recruitment to mount a strong Th1 immune response (16, 17). These observations together suggest that, BCG induces a sub-optimal immune response which is likely related to an inadequate activation of the APCs and subsequent priming of T cells during infancy even though, T cell mediated adaptive immune responses are critical for pulmonary immunity against TB (18–20).

In our previous studies, we sought to increase the efficacy of the BCG vaccine through recombinant technology. We identified a unique defect during the antigen processing of the BCG vaccine (21, 22). After BCG vaccination, APCs present antigens to CD4 and CD8 T cells through the MHC-I and MHC-II pathway, respectively, which mediate Th1 immunity. MHC-II dependent activation of CD4 T cells requires peptides from lysosomal degradation of BCG antigens (23). Paradoxically, BCG vaccine sequesters within the immature phagosomes of APCs avoiding lysosomal fusion, thereby reducing the MHC-II dependent CD4 T cell activation (24). This is because, BCG secretes *sapM* phosphatase which prevents lysosomal localization (25). In addition, we demonstrated that the near neutral pH of the BCG phagosomes prevents the activation of cathepsins and cathepsin-dependent *in situ* generation of an Ag85B-derived p25 immunogenic peptide (21). To bypass this defect we overexpressed the immunodominant secreted Ag85B in BCG, which triggered autophagy in APCs, increased lysosomal delivery, enhanced antigen processing and boosted vaccine efficacy against tuberculosis in mice (26). However, recombinant BCG vaccines still face two hurdles to enter into clinical trials; first, they need to go through safety and regulatory certification, and the infrastructure for the BCG vaccine manufacture and quality control which are established around the world would need to be adapted for production of recombinant BCG vaccines.

We therefore sought to increase the efficacy of the BCG Pasteur parent vaccine strain using novel adjuvants since, infant vaccines already contain adjuvants like alum (27). Because BCG vaccine phagosomes evade lysosomal degradation, we have investigated the hypothesis that agonists of the nucleotide-binding oligomerization domain-containing protein 2 (NOD2) and Retinoic acid-inducible gene-I (RIG-I) can act as adjuvants for BCG. Supporting a role for NOD2 adjuvants, previous studies show that mycobacterial cell-wall derived muramyl-dipeptide (MDP) activates NOD2 and it was used as an adjuvant for many subunit vaccines. The mycobacterial MDP is N-glycosylated unlike N-acetylated MDP from other bacteria and it is better in inducing cytokine secretion from MΦs (28). Coincidentally, the hypoimmunogenic Freund’s adjuvant also contains MDP (29). NOD2 plays a major role during pathogen sensing by phagocytic cells and determines the differential growth of BCG vaccine and MTB in MΦs (30–34). Together, these observations indicate that NOD2 signaling is an attractive target to boost immunity. Similar to NOD2, RIG-I, which senses bacterial and viral nucleic acids in the cytosol, is critical for initiating effective host immune responses against intracellular infections (35–37). Coincidentally, we had developed several novel small molecule nucleotide hybrid (SMNH^®^) - based agonists that ligate with NOD2 as well as RIG-I. We have reported that the dinucleotide SB 9200 (Inarigivir) activates RIG-I and NOD2 to provide a broad-spectrum antiviral activity against hepatitis B and hepatitis C viruses, as well as, Norovirus, RSV, influenza and parainfluenza (38–40).

In this study, we have tested the hypothesis that SMNH activation of NOD2/RIG-I, which are cytosolic sensors of pathogens, enhances the lysosomal delivery for the BCG vaccine increasing its peptide epitope production and thereby, T cell activation. Our studies show that Inarigivir and its derivative compounds increase the ability of APCs to activate T cells *ex vivo* during BCG infection. We further demonstrate that Inarigivir boosts the efficacy of the BCG vaccine against tuberculosis in mice and represents a new generation of adjuvants that target NOD2/RIG-I signaling and perhaps they can be safely combined with other vaccines.

## Materials and Methods

### Compounds

Inarigivir, a compound derived from the SMNH^®^ platform, is a dinucleoside phosphorothioate ester prodrug that is converted to the active dinucleoside phosphorothioate SB 9000 by esterase-mediated hydrolysis *in vivo*. Both Inarigivir and SB 9000 are a mixture of two isomers designated as Rp, and Sp and the chemical structures of the various compounds used in the study along with the details of their synthesis, and metabolism have been described elsewhere (38–40).

### Demonstration of SB 9200 (Inarigivir) Binding to RIG-I

*Sandwich ELISA Method*: A sandwich ELISA was developed for assessing the binding of SB 9000 (Inarigivir) to RIG-I by using SB 9000 derivatized with biotin at the 5′-OH group of the dinucleotide. Briefly, 96-well ELISA microplate was coated with anti-DDK antibody (*Origene, TA50011-100*) @ 1:200 dilution in coating buffer (0.1M carbonate, pH 9.6). 1 µg/ml DDK-tagged RIG-I recombinant protein (*Origene*, TP317615) was then captured onto microplate followed by blocking with superblock *(Thermofisher, 37537).* Biotinylated SB 9000 was then added at different concentrations and the SB 9000 bound to RIG-I, was detected using HRP-conjugated Streptavidin (*Origene*, AR100017) followed by development with TMB substrate solution (Thermofisher, 34028), followed by absorbance measurement at 450nm.

*Surface Plasmon Resonance Method:* For this assay, full length hRIG-I was generated in mammalian cells using Expi293F cells *(ThermoFisher Scientific, A14527)*, ExpiFectamine™ 293 Transfection Kit *(ThermoFisher Scientific, A14524)* and DDX58 - HaloTag(R) human ORF in pFN21A (Promega). The protein was then purified using HaloTag^®^ Mammalian Protein Purification System *(Promega, G6790)* according to manufacturer’s instructions. To perform SPR, various concentrations of biotinylated SB 9000 dissolved in water were manually printed onto PlexArray Nanocapture Sensor Chip (Plexera Bioscience, Seattle, WA) at 40% humidity. The signal changes after binding and washing (in AU) were recorded as the assay value. Selected protein-grafted regions in the SPR images were analyzed, and the average reflectivity variations of the chosen areas were plotted as a function of time. Real-time binding signals were recorded and analyzed by Data Analysis Module Kinetic analysis using BIA evaluation 4.1 software.

### Mycobacterial Culture and Reagents

Log-phase organisms of wild type *M. tuberculosis* H37Rv (ATCC 27294), *M. tuberculosis* Erdman (ATCC 35801) and *M. bovis* BCG (Pasteur strain,ATCC 35734) cultured in Middlebrook 7H9 broth for 7 days were frozen in aliquots. Before use, aliquots were thawed, washed three times in PBS (12,000 rpm; 15 min), sonicated at 4 watts using a soniprobe (60S Sonic Dismembrator, Fisher Scientific). A dispersed suspension adjusted to McFarland #1 in turbidity (10^8^ CFU/ml) was prepared for *in vitro* use as well as *in vivo* infection. *M. tuberculosis* H37Rv strain was used for *in vitro* infections whereas, *M. tuberculosis* Erdman strain was used for infection of mice *in vivo. M. bovis* BCG Pasteur strain (ATCC 35734) was used for all the *in vitro* as well as *in vivo* studies. For infection of MΦs and DCs *in vitro*, a multiplicity of infection (MOI) 1 was used uniformly in all assays confirmed by plating for CFUs on 7H11 agar. Inarigivir and other SMNH compounds were prepared in DMSO at concentration of 1 mg/ml and diluted as required. Doses used were well below cytotoxicity levels.

### Isolation and Cultivation of Primary Mouse MФs and DCs

Bone marrow from C57BL/6 mice (M/F) (5–6 weeks) (Jackson research Labs, USA) was obtained by flushing both the femur and tibia. The bone marrow pooled from two to four mice at a time was centrifuged for 5 min at one thousand rotations per minute. The bone marrows were washed two times in ACK lysing buffer (Fisher BW10-548E), followed by 1 ml of phosphate buffered saline (PBS) passed through a 27-gauge needle and suspended in Iscove’s Modified Dulbecco’s Medium (IMDM) medium supplemented with 10% percent fetal bovine serum (FBS), 20 ng per/ml mouse-granulocyte macrophage colony stimulating factor (GM-CSF) (for MФs), and 10 ng per/ml mouse-IL-4 (for DCs) and 10 µg/ml penicillin and gentamicin. The cells were then plated into six well plates and incubated at 37°C, 5% CO2 for 1 week, while adding the wells with two milliliters of fresh media, every two days. After seven days, the wells are flushed and the cells were pelleted and resuspended in MACS buffer; PBS containing 0.5% FBS, at a volume of 400 µl per 10^8^ cells. CD11c microbeads (Miltenyi 130-052-001; for DCs) or CD11b microbeads (for MФs) were then added to the cells, 100 µl per 10^8^ cells. The cells were incubated at 4°C for 15 min, washed once in 1 ml of MACS buffer and resuspended into 500 µl MACS buffer. During this time, the Miltenyi separation columns (Miltenyi 130-042-201) were prepared by rinsing once with 500 µl MACS buffer, one column per 10^8^ cells and placed onto magnetic holders. The cells were added to the columns and the flow through was collected. The columns are washed three times with 500 µl MACS buffer and then removed from the magnetic holders. One milliliter dissociation buffer was then flushed through the column. Wash off was repeated once. The cells (DCs and MΦs; APCs) were counted and plated into twenty-four well plates with 10^6^ cells per well in IMDM supplemented with 10% FBS and GM-CSF and allowed to differentiate for 4 to 7 days. Medium was then replaced with GM-CSF free medium and cells were used for various assays.

### *In Vitro* Antigen-85B Presentation Assay

This has been described in detail elsewhere, and the original method described by the Harding lab has been extensively used by us and others for *in vitro* antigen presentation by APCs (21, 22, 26, 41). Briefly, MTB-infected mouse APCs were washed after a 4-h infection and overlaid with the BB7-CD4 T cell hybridoma (a gift from Dr. David Canaday) which recognizes the Ag85B-p25 epitope in context of mouse MHC-II. IL-2 secreted from hybridoma T cells were determined using a sandwich ELISA kit (Ebiosciences). To test the effect of Inarigivir and other SMNH compounds on antigen presentation, APCs were pre-activated overnight at indicated dose, prior to infection with BCG. Doses used were well below cytotoxicity levels. Where indicated, the APCs were pharmacologically blocked with various inhibitors at indicated doses. Viability of APCs was monitored at >90% using trypan blue assay throughout the assay period.

### Cytokine assay in APCs

Quantification of cytokines produced by MΦs and DCs was carried out by enzyme-linked immunosorbent assay (ELISA) using commercially available kits (Biolegend for mouse IL-12, TNF-α IFN- β and IL-1β). Supernatants were collected at indicated times after activation or blockade with Inarigivir, SMNH compounds, other pharmacological agents followed by infection with MTB. Supernatants were first filtered through a 0.22-µm filter (EMD Millipore, MA, USA) before they were titrated for cytokine levels according to the manufacturer’s protocol.

### *M. tuberculosis* Growth Assay in MФs

Primary mouse MΦs, and human THP-1 MΦ cell line were lysed with 0.05% SDS at different time-points post MTB infection with or without Inarigivir and other pharmacological agents. Lysates were plated at serial 10-fold dilutions in PBS using 7H11 Middlebrook agar plates (Difco Laboratories, Surrey, UK). The plates were incubated at 37° for 3 weeks before counting colony-forming units (CFUs). Data were expressed as log10- CFUs per million APCs.

### Mouse Vaccination and Infection Experiments

Mouse models of vaccination used in this study have been previously described in detail (17, 26). Mice (C57Bl/6, M/F, 6–8 weeks of age; 20 per group) were immunized with BCG (Pasteur strain) given once subcutaneously at 1 x 10^6^ CFU per mouse or mixed with 25 μg of Inarigivir or its isomers. An experiment also included muramyl dipeptide (MDP; 25 μg) mixed with BCG vaccine and used as a combination vaccine for comparison. Four weeks later, mice were exposed to an aerosol dose of MTB (Erdman strain)(200- CFUs per mouse), using a Glas-Col (Indiana, USA) aerosol chamber. Mice were then housed in BSL3 conditions as per approved IACUC protocols, sacrificed 4 weeks later and their organs were processed for analysis of CFU and T cells in lungs and spleen as detailed below. For re-challenge studies shown in **Figure 7**, the primary challenge of animals was same as described above but mice were treated with INH (25 mg/kg) and rifampin (10 mg/kg) given orally as 15 daily doses over 3 weeks followed by resting until 4th week. Mice were then aerosol rechallenged with approximately 200 CFU of virulent MTB (Erdman strain) using a Glas-Col aerosol apparatus. Four weeks after rechallenge, organs were harvested for CFUs and T cell profiles in the lungs and spleens. Briefly, lungs and spleens were homogenized in 5 ml saline with 0.05% Tween 80 and 100 µl aliquots were then plated in replicates on 7H11 agar plates with 5 µg/ml thiophene 2-carboxylic acid hydrazide (TCH) that inhibits BCG. Plates were read for CFU counts of MTB and plotted against different vaccine groups. Vaccine induced-protection was expressed as log-10 reduction in CFU counts and significance was determined using two-way ANOVA with Dunett’s test. From one half of spleen of each mouse, T cells were fractionated and analyzed for IFN-γ secreting CD4 and CD8 T cells, tetramer stained CD4 and CD8 T cells and surface markers of memory using standard procedures (26). PE-labeled tetramer specific for Ag85B (CD4), and CD8 T cell tetramers specific for MTB32, ESAT6 and TB10.4 were prepared by the NIH tetramer facility at Emory University, USA. The short living effector CD4 T cells (SLECs) were typed as CD62L^-^ CCR7^−^ CD127^−^ CD44^+/−^ and memory precursor effector CD4 T cells were typed as (MPEC) as CD62L^+^CCR7^+^ CD127^+^/CD44^+/−^.

Significance of differences between groups was determined using the two-way ANOVA with Dunett’s post-hoc test. For each experiment, 10 mice per group were used, and 5 of them were used for CFU assays and three to five mice were used for analysis of various T cell populations in organs *via* flow cytometry staining. The data presented are an average of 2 independent experiments (each experiment having 10 mice per group (total 20 mice per group).

## Results

### SB 9000 (Inarigivir), the Active Metabolite of Inarigivir Shows Dose-Dependent Binding to Human RIG-I (hRIG-I)

**Figure 1:**
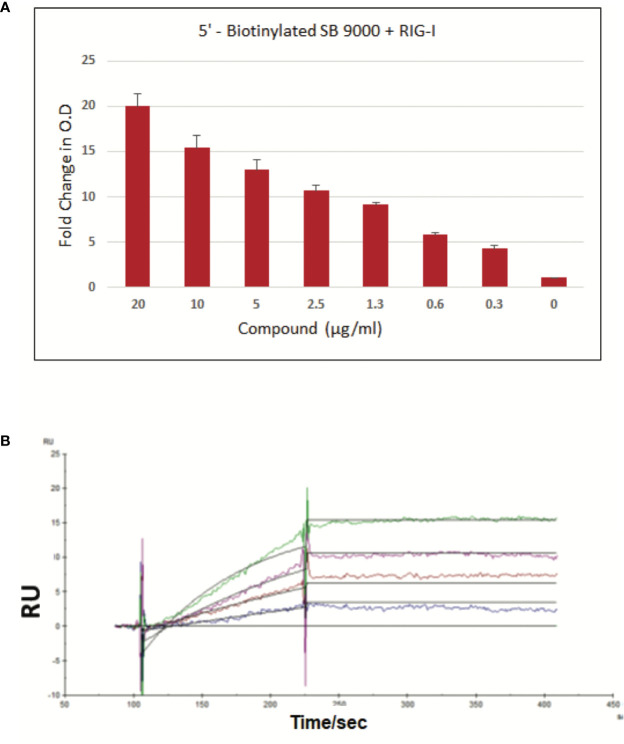
Binding of SB 9000 (Inarigivir) to retinoic acid-inducible gene I (RIG-I). **(A)** *Sandwich ELISA method*: Binding of SB 9000 to RIG-I by using SB 9000 derivatized with biotin at the 5′-OH group of the dinucleotide. ELISA microplate coated with anti-DDK antibody at 1:200 dilution in coating buffer (0.1M carbonate, pH 9.6), followed by 1 µg/ml DDK-tagged RIG-I recombinant protein. Biotinylated SB 9000 was then added as indicated and the SB 9000 bound to RIG-I was detected using HRP-conjugated Streptavidin followed by development with TMB substrate solution and absorbance measurement at 450nm (mean ± SD; n = 2). **(B)** *Surface Plasmon Resonance Method*: Human RIG-I (hRIG-I) was generated in mammalian cells using Expi293F cells *and* DDX58 - HaloTag(R) human ORF in pFN21A. The protein was then purified using HaloTag^®^ Mammalian Protein Purification System. SPR was measured by using various concentrations of biotinylated SB 9000 dissolved in water manually printed onto PlexArray Nanocapture Sensor Chip at 40% humidity. The signal changes after binding and washing (in AU) were recorded as the assay value. Selected protein-grafted regions in the SPR images were analyzed, and the average reflectivity variations of the chosen areas were plotted as a function of time. Real-time binding signals were recorded and analyzed by Data Analysis Module. Kinetic analysis was performed using BIA evaluation 4.1 software. Data are representative of 2 independent experiments, carried out in duplicates and values are expressed as mean ± SD.

To demonstrate a dose-dependent binding of SB 9000, a biotinylated form was synthesized by incorporating biotin at the 5′-OH group of SB 9000 *via* phosphoramidite chemistry. DDK-tagged RIG-I recombinant protein (*Origene*, TP317615) was then captured onto microplate and biotinylated SB 9000 was then added at different doses. Biotinylated SB 9000 bound to RIG-I, was detected using HRP-conjugated Streptavidin (**Figure 1A**). Dose-dependent binding of biotinylated SB 9000 to hRIG-I was clearly visible through ELISA based detection method, confirming that SB 9000 interacted with hRIG-I. We also found that SB 9000 binds to hRIG-I *via* its helicase domain (manuscript under preparation). In order to determine the Kd value of binding of Inarigivir to hRIG-I, surface plasmon resonance technique was further employed. The Kd value of binding was assessed to be 12 picomolar, demonstrating high affinity binding of SB 9000 to hRIG-I (**Figure 1B**).

### Inarigivir and Isomers Enhance *In Vitro* Antigen Presentation and Th1 Cytokine Production in MΦs

**Figure 2:**
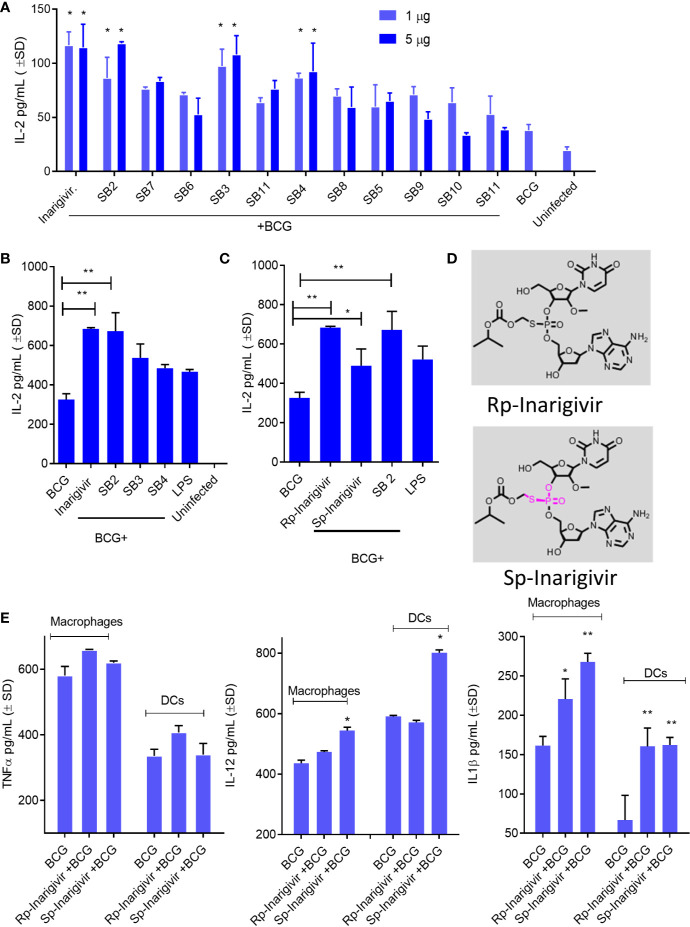
Small molecule nucleotide hybrids (SMNHs, aka. SB series) enhance antigen presentation by BCG vaccine infected macrophages and increase Th1 cytokine secretion. C57Bl/6 mouse bone marrow derived MΦs and dendritic cells (DCs) (aka. APCs) were treated with SMNHs (SB series) at indicated doses, followed by infection with *Mycobacterium bovis* Bacille Calmette Guerin (BCG) for 4 h (MOI = 1). Mycobacterial Antigen-85B (Ag85B) derived p25 peptide specific BB7 CD4 T cells were overlaid and supernatants collected after 18 h were tested for IL-2 using sandwich ELISA. **(A)** Initial screening of SB series of SMNHs including Inarigivir (NOD2/RIG-I agonist), SB-2 (TLR-7/9 agonist), and SB-3 and SB-4 (dinucleotides related to Inarigivir). *p values vs. BCG alone. **(B**, **C)** Comparison of Inarigivir and its resynthesized isomers Rp-Inarigivir and Sp-Inarigivir (10 µM) with the LPS agonist of TLR-4 (1 µg/ml) and related SB compounds during antigen presentation. *p values for groups compared. **(D)** Structures of Rp-Inarigivir and Sp-Inarigivir. **(E)** MΦs and DCs were treated or not with Rp-Inarigivir and Sp-Inarigivir (10 µM), followed by BCG infection. Supernatants collected at 18 h tested for Th1 cytokine levels using sandwich ELISA. *p values for treated vs. BCG alone (*<0.01; **< 0.009; p values, 1-way ANOVA with Tukey’s posttest). Data are representative of two independent experiments carried out in duplicates and values are expressed as mean ± SD.

Adjuvants modulate cytokine secretion in APCs, reduce the dose of antigen required, sustain immune responses through T and B cell activation and increase vaccine efficacy, although individual agents vary greatly in their efficacy. To determine the adjuvant activity, Inarigivir and isomers were screened using an *in vitro* antigen presentation assay (AGPR) (21). APCs like MΦs and DCs present peptides through MHC-II and MHC-I pathway to activate CD4 and CD8 T cells respectively. Th1 immunity mediated by these T cells in turn, protects against intracellular infections including tuberculosis (23). The MHC-II pathway involves sorting the vaccine into lysosomes, where the Cathepsin-dependent degradation of the vaccine produces peptide epitopes (21). These are in turn, loaded into MHC-II -peptide complex which is then exported to the plasma membrane for activation of CD4 T cells. In contrast, BCG secreted Ag85B can leak into the cytosol to be digested by the proteasome and the peptides exported through a TAP-dependent process to MHC-I for the activation of CD8 T cells. An *in vitro* mycobacterial AGPR model has been described in which, APCs infected with BCG vaccine or MTB process and present the Ag85B-derived p25 epitope to a p25-specific BB7 CD4 T cell hybridoma (21, 22, 26, 41, 42). Upon recognition of the MHC-II-p25 complex, these T cells secrete IL-2, which is an indicator of antigen presentation. AGPR screening studies indicated an enhanced antigen presentation by MΦs when SMNH compounds were combined with BCG vaccine (**Figure 2A**). Four compounds (Inarigivir, SB-2, SB-3 and SB-4) that induced significantly higher antigen presentation were identified of which, Inarigivir was more potent compared to other SB compounds while taking into consideration the effective doses. Inarigivir was then further compared with other SB compounds that activate TLR-7/9 (unpublished) along with TLR-4 activating LPS to determine its relative adjuvant effect. LPS is a known agonist of TLR-4, and hence was used as a positive control. While SB2 was also found to be as effective as Inarigivir in inducing antigen presentation, SB-3, SB-4 and LPS induced significantly less antigen presentation in MФs (**Figure 2B**). It is important to note here that Inarigivir was able to induce better antigen presentation as compared to SB2 compound even at a lower dose of 1 µg/ml (**Figure 1A**). Since Inarigivir is a mixture of two isomers, it was of interest to determine if a single isomer had more potent adjuvant activity. The isomers were therefore synthesized and compared with LPS (**Figures 2C–D**). We found Rp isomer of inarigivir to be more effective in inducing antigen presentation in MΦs as compared to Sp isomer. However, when we examined the effect of these isomers on Th1 cytokines secreted by MΦs and DCs, both these compounds were equally potent in inducing IL-12 and IL-1β in MΦs and DCs, although some variability was seen between these 2 cell types (**Figure 2E**). Neither Rp or Sp isomer combination with BCG induced significant levels of TNF-α when compared with BCG alone.

### NOD2-/RIG-I Agonist Inarigivir and Its Isomers Increase Caspase-1–Dependent IL-1β Secretion and NOD2-Dependent Antigen Presentation

**Figure 3:**
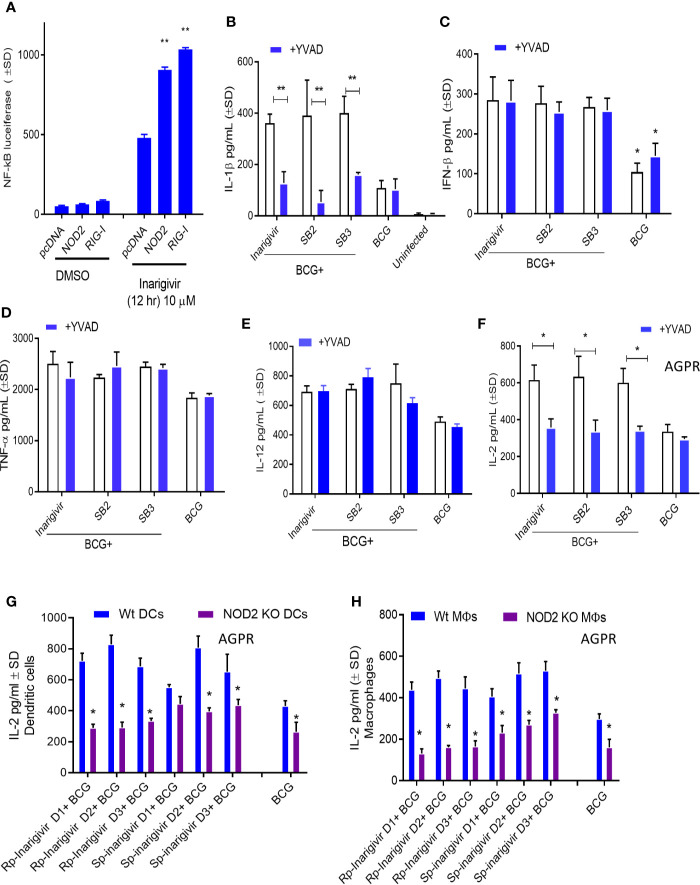
Inarigivir activates NOD2/RIG-I receptors enhancing Capsase-1 dependent IL-1β secretion and NOD2 dependent antigen presentation. HEK cells were used for assay of receptor activation **(A)** and wild type C57Bl/6 **(B–F)** or NOD2^−/−^ **(G–H)** mouse derived APCs were left either untreated or treated with Inarigivir, Rp-Inarigivir and Sp-Inarigivir and SB-2 or SB-3 (10 µM), followed by inhibitors as indicated, and where indicated, a 4-h infection with BCG (MOI = 1). Supernatants were tested for cytokines at 18 h or APCs overlaid with BB7 CD4 T cells for antigen presentation assay to measure IL-2. **(A)** Induction of NF-kB activity by Inarigivir (mix of Rp-Inarigivir and Sp-Inarigivir) through NOD2 and RIG-I activation. HEK-293 cells were transfected with pcDNA, NOD2, RIG-I, and NF-kB-luciferase. The cells were then incubated with *Inarigivir* (10 µM). Following 12 h incubation, Luciferase activity was measured and presented as mean ± S.D. from three independent experiments. **(B)** Rp-Inarigivir and Sp-Inarigivir combinations with BCG increase IL-1β secretion which is decreased after Caspase-1 blockade using Z-YVAD-fmk (50 µM). **(C–E)** IFN-β, TNF-α, and IL-12 cytokines are not affected by the Caspase-I blockade, although Inarigivir, SB2 and SB3 enhance IFN-β levels. **(F)** Caspase-1 blockade decreases antigen presentation. **(G, H)** Rp-Inarigivir and Sp-Inarigivir enhance antigen presentation by BCG infected wt-APCs, which is decreased in NOD2^−/−^ APCs. (* < 0.01 ** < 0.006 p values using 1-way ANOVA with Tukey’s posttest). Data are representative of 2 independent experiments carried out in duplicates and values are expressed as mean ± SD.

Additional studies were carried out to confirm NOD2 and RIG-I receptor activation by Inarigivir using live cell cultures. Increased NF-κB luciferase activity in HEK-293 cells after transfection with NOD2, RIG-I and NF-κB, was observed only when they were incubated with Inarigivir, which confirmed activation of these receptors (**Figure 3A**). Since NOD2 protein is also a part of some inflammasomes which can trigger an intracellular inflammatory response (43), it was of interest to see if inflammasome activation could have been triggered by activation of NOD2 by Inarigivir. It is known that NOD2 containing inflammasomes (aka. *Nodosomes*) mediate the cleavage of IL-1β through Caspase-1 (44, 45). Thus IL-1β secretion by MΦs with and without Inarigivir addition was measured during BCG infection and concurrently, caspase-1 specific inhibitor Z-YVAD-fmk was added to another set of MΦs (**Figure 3B**). Z-YVAD–fmk, which is an irreversible inhibitor of Caspase-1, can prevent the downstream activation of inflammasome signaling by preventing the cleavage of pro-IL-1β into active secreted form of IL-1β (46). Increased production of IL-β by MΦs that received Inarigivir and BCG compared to MΦs that received BCG only, indicated the activation of inflammasome by Inarigivir. Blockade of Caspase-1 using Z-YVAD-fmk abrogated this increased IL-1β secretion to a significant extent, which further confirmed that, Inarigivir mediated activation of NOD2 led to the secretion of IL-1β through inflammasome signaling. SB2 and SB3, that ligate with TLR-7 and TLR-9 also induced IL-1β secretion *via* inflammasome activation since blockade of Caspase-1 with Z-YVAD-fmk resulted in a significant reduction of this cytokine.

Apart from NOD2 activation, Inarigivir activates RIG-I receptor, which is known to trigger intracellular interferon (IFN) response (47). For example, we showed that Inarigivir and isomers activate RIG-I, and (a) its Interaction with RIG-I led subsequent launching of a type I IFN response, and (b) its binding to RIG-I resulted in the direct inhibition of viral replication (38–40). In order to determine if Inarigivir mediated activation of RIG-I triggers type I IFN response during BCG infection as well, IFN-β levels secreted by MΦs after addition of Inarigivir were measured during BCG infection (**Figure 3C**). IFN-β levels were found to be markedly increased in MΦs that received Inarigivir as well as BCG compared to MΦs that received only BCG. Addition of Z-YVAD-fmk did not alter the levels of IFN-β in MΦs during BCG infection with and without Inarigivir and other SMNH compounds, which indicated that IFN-β response was independent of inflammasome activation. Increased IFN-β levels were also seen in MΦs that were activated with SB2 and SB3 compounds during BCG infection, and in MΦs that were treated with Z-YVAD-fmk, suggesting that the activation of TLR-7/9 also induces IFN-β response in an inflammasome independent manner.

To determine the specificity of inflammasome activation, supernatants of APCs activated with SMNH and BCG with or without Z-YVAD-fmk were titrated for proinflammatory cytokines TNF-α and IL-12. The levels of TNF-α and IL-12 were significantly higher in MΦs that received Inarigivir, SB2 and SV3 compounds compared to MΦs that received only BCG **(** **Figures 3D, E**). However, Z-YVAD-fmk had no significant effect, suggesting that induction of TNF-α and IL-12 by Inarigivir was independent of inflammasome activation.

Since some of the cytokines (IFN-β, TNF-α and IL-12) were induced in inflammasome independent manner whereas, IL-1β was inflammasome dependent, it was of interest to find out if the enhancement of antigen presentation by Inarigivir was dependent or independent of inflammasome activation. MΦs were infected with BCG combinations with Inarigivir, SB2 and SB3 followed by AGPR for IL-2 levels using BB7 hybridoma T cells in the presence and absence of Z-YVAD-fmk (**Figure 3F**). Increased levels of IL-2 induced by Inarigivir were abrogated by Z-YVAD-fmk, indicating that enhance antigen presentation was dependent on inflammasome activation. This effect was also observed with SB2 and SB3 compounds, indicating that TLR-7/9 activation mediated increased antigen presentation also involved inflammasome activation.

To further confirm that NOD2 activation is involved in the Inarigivir mediated enhanced antigen presentation, we examined the antigen presentation using MΦs and DCs derived from the bone marrow of WT and NOD2 knockout mice. NOD2 KO MΦs and DCs showed markedly reduced levels of antigen presentation indicated by IL-2 levels measured daily over 3 days in presence of either Rp or Sp isomers of Inarigivir compared to those from wt-mice (**Figures 3G, H**). These data confirm that Inarigivir enhances antigen presentation *via* NOD2 activation.

### Inarigivir Enhances the *In Vitro* Bactericidal Activity of MΦs Against *M. tuberculosis*

**Figure 4:**
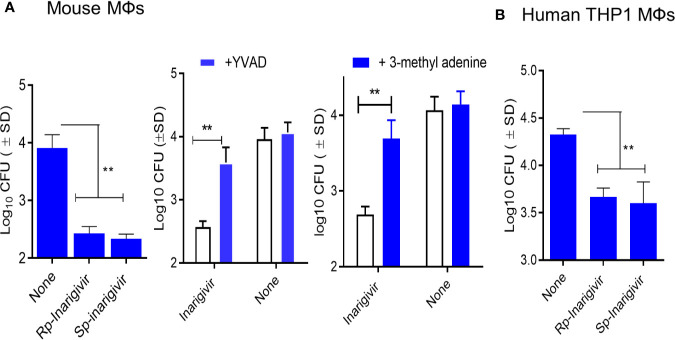
Inarigivir enhances the bactericidal function of mouse and human THP-1 macrophages against *M. tuberculosis*. Wt-C57Bl/6 mouse derived MΦs and phorbol myristyl acetate activated THP-1 human MΦs were treated with Rp-Inarigivir and Sp-Inarigivir or Inarigivir (10 µM) followed by infection with *M. tuberculosis* and incubated maintaining >90% viability of MΦs (MOI = 1). On day three, lysates were plated for colony counts (CFUs) of MTB. **(A)** Rp-Inarigivir and Sp-Inarigivir enhance the bactericidal function mouse derived MΦs, which is decreased by prior incubation of MΦs with either Z-YVAD-fmk inhibitor of Caspase-1 (50 µM) or 3-methyladenine (100 µM), which inhibits autophagy through PI-3 Kinase. **(B)** Rp-Inarigivir and Sp-Inarigivir enhance the bactericidal function THP-1 MΦs. (**p < 0.007; 1-way ANOVA with Tukey’s posttest). Data are representative of 2 independent experiments carried out in duplicates and values are expressed as mean ± SD.

We had earlier observed that TLR-4 activation of mouse MΦs through LPS led to an increased bactericidal activity against mycobacteria through the induction of autophagy (48). NOD2 signaling pathway is also linked to the induction of mycobacterial autophagy (49). To determine if NOD2/RIG-I activation enhances the bactericidal activity, mouse and THP1 human MΦs were activated *in vitro* with *Rp*-inarigivir and *Sp*-inarigivir, followed by infection with viable MTB and growth assays. MΦs were blockaded using Z-YVAD-fmk or 3-methyladenine to determine the role of Caspase-1 and autophagy, respectively. Prior activation of both mouse and human MΦs with Inarigivir led to a significant decline in the CFU counts of intracellular MTB **(** **Figures 4A, B**). Caspase-1 and autophagy blockade *via* Z-YVAD and 3-MA markedly reduced the Inarigivir mediated bactericidal activity in mouse and MΦs respectively, suggesting that it promotes intracellular killing of MTB through inflammasome and autophagy dependent pathways (**Figure 4A**).

### Inarigivir Enhances the Efficacy of BCG Vaccine Against Experimental Aerosol-Induced Tuberculosis Through an Expansion of T Cell Functions

**Figure 5:**
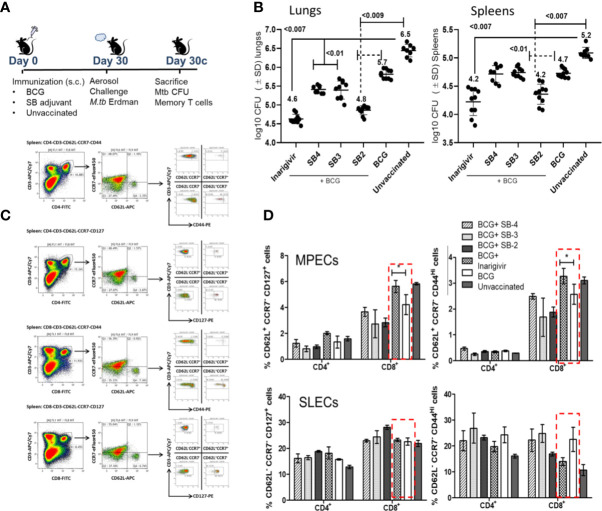
Inarigivir enhances the efficacy of BCG vaccine against aerosol induced tuberculosis of mice and induces an expansion of memory T cells. **(A)** C57Bl/6 mice were immunized subcutaneously with one dose of BCG (1 x 10^6^ CFU) alone or mixed with 25 µg per dose of Inarigivir or indicated SB compounds. Four weeks later, mice were aerosol challenged with of ~200 CFUs of *M. tuberculosis (*Erdman strain*)* in the lungs of mice and bacterial counts (CFUs) of lungs and spleens at indicated times were determined by plating their homogenates on 7H11 agar. Spleen derived T cells were analyzed using flow cytometry for memory markers. **(B)** Inarigivir-BCG vaccine combination is better than BCG combinations with SB-2, SB3 and SB4 in reducing the MTB burden of lungs and spleens of mice (10 mice per group; 5 mice each from two experiments; p values; 2-way ANOVA with Dunette’s *post hoc* test). Individual value of Log10 CFUs of organs for each mouse from 2 independent experiments are shown. **(C)** Histogram analysis of spleen derived T cells (n = 3 per group per experiment and represents the results of 2 independent experiments) for T cells using flow cytometry at the time of sacrifice. **(D)** Inarigivir-BCG vaccine combination induces higher levels of memory precursor-effector T cells (MPECs; CD62L^+^ CCR7^+^ CD127^+^ CD44^+/−^) and comparable short lived-effector T cells (SLECs; CD62L^−^ CCR7^−^CD127^+^CD44^+/−^) (* < 0.005, p values, *t* test). Data are representative of 2 independent experiments carried out in duplicates (after pooling 3 mice from per group, per experiment) and values are expressed as mean ± SD.

Our previous studies showed that a stronger *in vitro* AGPR by BCG infected APCs correlated with its better efficacy against tuberculosis in mice (17, 26). Thus, Inarigivir and the SB 2–4 compounds were mixed separately with the BCG vaccine (25 µg/dose/mouse) and used for immunization of mice, followed by an aerosol challenge with virulent MTB and evaluation of protection using bacterial counts, as described before (17). During this initial screening using the NIH mouse TB vaccine evaluation model (**Figure 5A**), as expected, BCG vaccine given alone protected mice by reducing the lung burden of MTB by ~1-log_10_ as compared to unvaccinated mice (**Figure 5B**). Whereas, SB-adjuvant combinations with BCG showed an enhanced protection, the NOD2/RIG-I activating Inarigivir generated the best protection (~2-log10 decline in CFUs of lungs and spleens vs. naïve mice; ~ 1-log10 better than BCG alone). Of note, SB2 was also protective as an adjuvant. Since Inarigivir and SB compounds alone had no effect on the growth of tuberculosis in mice (not shown), these data suggested a strong *in vivo* adjuvant action for Inarigivir and its isomers.

While it is well known that Th1 immunity controls tuberculosis in mice through key cytokines IL-12, TNF-α and IL1-β, they are variably expressed after BCG vaccination in mice. For example, BCG is a poor inducer of mature IL1-β in mouse MΦs unlike MTB (50). It is well known that BCG induces robust effector-memory CD4 T cell responses in mice, but less effective CD8 type CTLs (51, 52). Of note, BCG has also been reported as a poor inducer of central memory T cells (TCMs) in mice, and TCMs are thought to mediate long-term memory against tuberculosis (8). To correlate the enhanced protection generated by Inarigivir with the expansion of memory T cells, spleens of mice were analyzed for T cells expressing markers of memory precursor effector T cells (MPECs; CD62L^+^ CCR7^−/+^ CD127^+^ CD44^−/+^) and short lived effector T cells (SLECs; CD62L^−^ CCR7^−^ CD127^+^ CD44^−/+^); **Figure 5C** illustrates the gating strategy for memory T cells. Compared to BCG alone, Inarigivir combination with BCG did not have a significant effect on CD4 and CD8 T SLEC population (**Figure 5D**). However, Inarigivir BCG combination induced higher levels of CD8 T cell MPECs when compared with mice that received BCG alone or other adjuvants in combination. These data indicated that activation of NOD2 and RIG-I pathways *in vivo* could be important to boost CD8 memory T cells. The data also underscore the importance of memory CD8 T cells during protective immunity *in vivo* although, they not rule out the role of other T cell subsets or myeloid cells which remain to be explored after *in vivo* administration of Inarigivir during BCG vaccination.

### Inarigivir Combination With BCG Vaccine Protects Better Than Muramyl-Dipeptide–BCG Combination Against Tuberculosis in Mice

**Figure 6:**
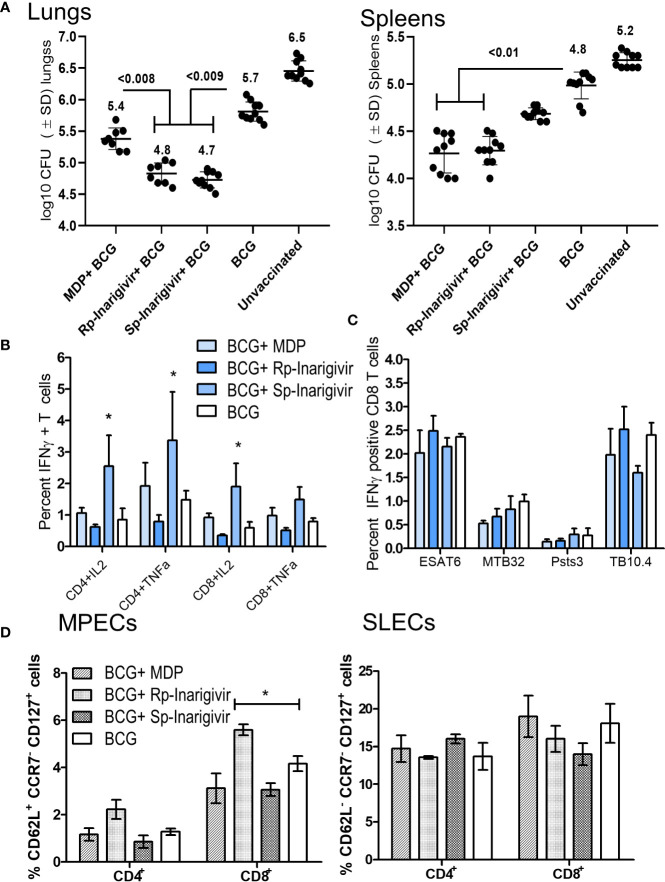
Rp-Inarigivir and Sp-Inarigivir are better adjuvants for BCG vaccine compared to muramyl dipeptide (MDP) in mice. **(A)** C57Bl/6 mice were vaccinated with BCG alone, BCG mixed with Rp-Inarigivir, Sp-Inarigivir (10^6^ CFU of BCG mixed with 25 µg/dose of adjuvants given once, s.c.) or MDP (25 µg/dose) followed by an aerosol challenge with *M. tuberculosis* (~200 CFUs/mouse) for evaluation of protection as in Fig. 4A. Rp-Inarigivir and Sp-Inarigivir combinations with BCG are more effective than BCG alone or BCG combination with MDP in reducing the growth of MTB in the lungs (5 mice per group per experiment; p values, 2-way ANOVA with Dunette’s *post hoc* test). Individual value of Log10 CFUs of organs for each mouse from 2 independent experiments are shown (10 mice per group; 5 mice each from two experiments). **(B, C)** Inarigivir and Sp-Inarigivir combinations with BCG induce better levels of cytokine positive T cells in the spleens (panel B) and comparable levels of BCG antigen specific CD8 T cell tetramers specific for ESAT6, MTB32, Psts, TB10.4 antigens (3 mice per group per experiment; * < 0.01 p values vs. BCG + MDP groups; 1-way ANOVA. Data are representative of 2 independent experiments carried out in duplicates (after pooling 3 mice per group per experiment) and values are expressed as mean ± SD. **(D)** Inarigivir and Sp-Inarigivir combinations with BCG induce better levels of MPECs in the spleens of vaccinated mice (3 mice per group; * < 0.01 p values; 1-way ANOVA). Data are representative of 2 independent experiments carried out in duplicates (after pooling 3 mice per group per experiment) and values are expressed as mean ± SD.

Data presented above indicated that Inarigivir and its isomers are potent adjuvants enhancing both the *in vitro* function of APCs and *in vivo* efficacy of the BCG vaccine in mice. The mycobacterial NOD2 ligand, muramyl-dipeptide (MDP) has been extensively tested as an adjuvant inducing IL-1β from MΦs, but moderately increasing the efficacy of the BCG vaccine in mice (53). Therefore, we sought to compare the activity of Inarigivir isomers with MDP to determine if they are more potent. Mice were vaccinated with BCG in separate combinations with MDP, *Rp*-inarigivir and *Sp*-inarigivir compounds followed by an aerosol challenge with MTB (**Figure 4A** **;** NIH model). While MDP-BCG combination was found as potent as *Rp*-inarigivir in reducing the bacterial burden in spleens of mice, both isomers of Inarigivir were found to be superior to MDP in providing better protection in lungs. These results suggest that combined activation of NOD2 as well as RIG-I pathways could generates a more protective immune response compared to the activation of only NOD2 pathway using MDP (**Figure 6A**). These data are also consistent with the finding that, a combination of TLR-2 and MDP dual adjuvant combination with BCG reduced the lung MTB burden by < 0. 5-log10 (53). Increased efficacy of *Rp*-Inarigivir isomer adjuvant was also reflected by a better induction of Th1 cytokine positive CD4 and CD8 T cells in the spleens (**Figure 6B**), though the difference was not found statistically significant when measured specifically for MTB antigen specific CD8 T cells. (**Figure 6C**). Comparison of MPECs and SLECs in different vaccination groups once again demonstrated an increased expansion of CD8 T MPECs by Inarigivir BCG combination that was superior to MDP-BCG combination **(** **Figure 6D** **).** Since levels of CD4 and CD8 SLECs was not significantly different among the different vaccination groups, we suggest that SLECs may be less important for Inarigivir mediated protective immune response against tuberculosis (**Figure 6D**). Together, these data emphasize the importance of combined activation of NOD2 and RIG-I pathway in expanding CD8 T MPECs.

### Inarigivir Combination With BCG Vaccine Enables Stronger Recall Immunity Against Tuberculosis

**Figure 7:**
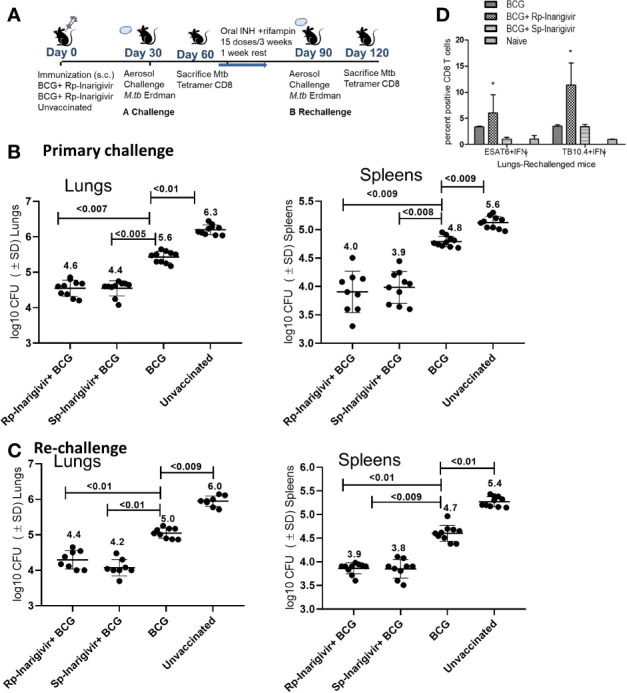
Inarigivir and Sp-Inarigivir combinations with BCG induce better T cell responses allowing mice to mount a better recall response against re-challenge with tuberculosis. **(A)** C57Bl/6 mice were vaccinated with Inarigivir and Sp-Inarigivir combinations with BCG and infected with MTB as in **Figure 4** for protection. As indicated, a separate group of challenged mice were cleared of vaccine and MTB organisms using isoniazid (25 mg/kg oral daily) and rifampin (10 mg/kg oral daily) for 3 weeks followed by resting for 1 week and re-challenged with an aerosol dose of virulent MTB (~200 CFUs per mouse). Four weeks later, protection was evaluated through CFU counts of lungs and spleens (10 mice per group; 5 mice each from two experiments) and day 120 lung T cell analysis (3 mice per group). **(B**, **C)** Rp-Inarigivir and Sp-Inarigivir combinations with BCG protect better than BCG both after primary and secondary rechallenge (p values, 2-way ANOVA with Dunette’s *post hoc* test). Individual value of Log10 CFUs of organs for each mouse from 2 independent experiments groups are shown. **(D)** Rp-Inarigivir combination with BCG increases the numbers of ESAT6^+^ IFN-γ^+^ and TB10.4^+^ IFN-γ^+^ CD8 T cells. (* p < 0.01, t test). Data are representative of two independent experiments carried out in duplicates (3 mice pools per group per experiment) and values are expressed as mean ± SD.

An interesting feature of tuberculosis in humans is that post-infection immunity may not be strongly induced to last long enough in some individuals because of which, reinfections occur. Indeed, BCG poorly protects mice after reinfection with tuberculosis (54). It is known from many mouse virus infection models that the longer living MPECs give rise to TCMs, which persist in low numbers but undergo a strong recall expansion into effector T cells (TEMs) upon pathogen chellenge and secrete Th1 cytokines, expressing perforin and granzyme to control reinfection (55–57). To validate the recall function of BCG induced MPECs, which were present in significant numbers after Inarigivir-BCG vaccination (**Figures 5** and **6**), a re-challenge of tuberculosis infection model was established (**Figure 7A** **).** Mice were vaccinated with Rp-inarigivir and Sp-inarigivir in combination with BCG, and a primary challenge with MTB was followed by a post primary challenge. One experimental set of mice was sacrificed after primary challenge for the examination of MTB CFUs in lungs and spleens. The mice of second set were treated with a mix of isoniazid and rifampin drugs for 3 weeks to eliminate both vaccine and MTB organisms as confirmed by CFU counts (data not shown). One week later, mice were re-challenged with virulent MTB and sacrificed 4 weeks later followed by MTB counts. Strikingly, *Rp*-inarigivir and *Sp*-inarigivir combination with BCG mounted an even better level of protection in both lungs and spleens after re-challenge of mice compared to BCG vaccine alone (**Figures 7B, C** **)**. Interestingly, during rechallenge, mice given *Rp*-inarigivir combination with BCG also showed increased level of antigen specific (ESAT6^+^ and TB10.4^+^) IFN-γ^+^ CD8 T cells in the spleens, which was not seen during the primary challenge (**Figure 7D** vs. **Figure 6C**). We suggest that, persistent tetramer + CD8 T cells from Inarigivir and BCG primary vaccinated mice (**Figure 6C**) showed a stronger recall expansion after MTB challenge (**Figure 6C**) indicating robust memory induction.

## Discussion

Adjuvants have been frequently used to boost the immunogenicity of the subunit vaccines but not for live attenuated BCG, since live BCG organisms can induce cell mediated and humoral immunity on their own (58, 59). Nonetheless, adjuvants for BCG may be required since it protects only partially against lung tuberculosis of children and adults (5, 6). A likely reason for the partial efficacy of BCG in children is the defective expansion of T cell immunity in neonates since the vaccine is given at birth when immune system is not fully developed (1). Indeed, a defect in the development of protective Th1-type of immune responses and a skewing toward Th2 immunity is a feature of the neonatal T-cell immunity (60). We suggest that this physiological defect is likely to predispose them to tuberculosis despite BCG vaccination. In addition, many epidemiological studies document that the neonatal system is physiologically immature as evidenced by the requirement of booster vaccinations for DPT and MMR among infants and an immune environment which is tolerant (61). Safer adjuvants like alum are already in use during infant vaccination, and in this study, we sought to determine whether the efficacy of the infant BCG vaccine can be augmented using novel adjuvants.

Intracellular PRRs that recognize foreign nucleic acids play an important role in host defense activating innate and adaptive immunity against microbial infection (62). Small synthetic analogs of microbial DNA and RNAs have been found to mimic immunostimulatory properties of intact microbes, allowing us to target nucleic acid sensing pathways and design novel adjuvants (63, 64). Through changes in their sugar phosphate backbone, synthetic oligonucleotides can provide the advantage of stability in biological tissues and fluids while retaining their native affinity to targets. One such small molecule nucleotide homolog, Inarigivir developed by us, enhanced host antiviral immune response against hepatitis B and C infections in our previous studies (38–40). Inarigivir is an orally bioavailable dinucleotide that activates the viral sensor proteins, RIG-I and NOD2 and triggers the induction of the interferon signaling cascade for antiviral defense.

IFNs play a major defensive role protecting against intracellular bacterial infections, including TB (65, 66). IFNs have a remarkable effect on monocyte derived MΦs and DCs although, type I and II IFNs differ in their effects on MФs. For example, IFN-γ can induce IL-12 which is required for a Th1 polarization of T cells and more importantly, activate nitric oxide to kill intracellular pathogens (2, 67, 68) In contrast, type I IFNs including IFN-α and IFN-β show both beneficial and inhibitory effects on MTB growth depending upon the mouse or human MФ phenotype (3). Interestingly, BCG induces less IFN-β in mouse DCs, and addition of recombinant IFN-β increased the immunogenicity of BCG infected DCs (4). The decreased ability of BCG to induce IFN-β is likely due to the absence of the ESX-1 secretion system, which allows the leakage of nucleic acids from BCG phagosomes to cytosol for activation of nucleic acid sensors (69). Indeed, MTB which has an intact ESX-1 system, leaks DNA from its phagosomes activating a variety of cytosolic sensors triggering IFN-β (5).

These considerations led us to first screen a library of SMNHs for their ability to boost antigen presentation in BCG infected MФs or DCs because it is a robust technique requiring the lysosomal degradation of the BCG vaccine, followed by a peptide epitope export through MHC-II pathway for T cell activation (**Figure 1**). Because BCG evades lysosomal fusion through a *sapM-*dependent mechanism (25), our first goal was to determine whether SMNH adjuvants enhance lysosomal degradation of BCG vaccine. Using AGPR and cytokine assays, we identified that the lead compound Inarigivir was the most active compound enhancing not only *in vitro* antigen presentation but also secretion of IFN-β and Th1 cytokines including IL-1β (**Figures 1**–**3**). Inarigivir induced robust AGPR in both BCG infected MФs and DCs and was better than other SMNH analogs or the TLR-4 agonist LPS, when doses and sustainability of antigen presentation were considered (**Figures 2A–D** and **Figures 3F–H**). Our previous studies show that increased lysosomal localization of recombinant BCG and MTB derived vaccines lead to robust Ag85B epitope presentation to CD4 T cells *in vitro* (21, 26). This study shows that SMNH adjuvant activation has a similar effect on BCG Pasteur vaccine.

Intriguingly, Inarigivir-boosted AGPR in APCs was dependent on Caspase-1 and NOD2 indicating a novel molecular mechanism for this adjuvant (**Figures 3F–H**). Our data are consistent with the observation that NOD2 and TLR2 co-signaling enhance antigen processing (70, 71). Furthermore, lysosomal degradation of BCG is essential for AGPR, and therefore, Inarigivir mediated activation of NOD2 and RIG-I signaling appears to have bypassed the *sapM-*dependent evasion of antigen processing by BCG infected APCs (72, 73).

Because Inarigivir treated BCG underwent lysosomal degradation, we hypothesized that the adjuvant can also have a similar effect on MTB in MФs. **Figure 4** illustrates that, Inarigivir improved the clearance of intracellular MTB in both mouse and human MФs. Autophagy was found to be important for the intracellular clearance of MTB through Inarigivir, since blockade of autophagy by 3- methyl adenine abrogated the antimicrobial effect (**Figure 4B**). In addition, blockade of caspase-1 also reduced the Inarigivir mediated antimicrobial effect in macrophages, suggesting that a combination of NOD2 and RIG-I dependent mechanisms increase the antimicrobial function of MФs. These data are consistent with the observation that NOD2 and RIG-I receptor stimulation induces autophagy, boosts antibacterial effect and antigen presentation, although not in the context of tuberculosis (74, 75). Notably, autophagy is linked with inflammasome activation which requires caspase-1, and excessive activation of inflammasome triggers its degradation *via* autophagy (45, 46). Finally, capsase-1 enhances the acidification of phagosomal lumen through the activation of NOX2-phagocyte oxidase (75). We demonstrated earlier that that cathepsin-D cleaves Ag85B producing the p25 immunogenic epitope during antigen presentation which requires lysosomal acidification through vATPpase (21). Combining these observations, we propose that Inarigivir likely induces a combination of NOD2 and RIG-I dependent mechanisms to increase the immunogenicity of the BCG vaccine in APCs.

The enhanced *in vitro* immunogenicity of Inarigivir and BCG-infected APCs (**Figures 1** **–** **3**) suggested that the combination would also generate better *in vivo* immune responses. **Figures 5** to **7** validate this observation. The NIH model for tuberculosis vaccination shows that the protective efficacy of the BCG Pasteur vaccine is measured by a decrease in the lung burden of MTB organism by ~1-log10 CFUs compared to unvaccinated mice. **Figure 5** shows that Inarigivir and BCG vaccination combination induced a ~1-log10 higher decline in MTB counts compared to BCG vaccine alone or its combination with other SMNH analogs. The spleens of Inarigivir and BCG vaccinated mice showed an increase in the levels of CD8 MPEC T cells, while also maintaining comparable levels of SLEC-T cells induced by the BCG vaccine (**Figure 5**). Since MDP is also a well-known NOD2 agonist, both *Rp*-Inarigivir and *Sp*-Inarigivir combinations with BCG were compared with MDP and BCG combination. In a recent study, BCG vaccine combination with MDP showed < 0.5 log10 reduction of MTB burden in the lungs of mice (53). In contrast, both *Rp*-Inarigivir and *Sp*-Inarigivir were superior to MDP in decreasing the lung burden of MTB by ~1-log10 (**Figure 6**). Furthermore, when compared to MDP-BCG combination, *Rp*-Inarigivir-BCG combination was superior in increasing the levels of cytokine positive T cells and CD8 T MPECs in spleens. Additional studies using re-challenge experiments showed that such MPECs persisted much longer in mice that were vaccinated with Inarigivir BCG combination and presumably protected them against reinfection with MTB (**Figure 7**). Furthermore, *Rp-*Inarigivir and *Sp*-Inarigivir combinations with BCG protected mice against tuberculosis both after primary and re-challenge infections suggesting that Inarigivir, which is mixture of the RP and SP isomers is likely one of the most potent adjuvants for BCG vaccine reported to date.

In conclusion, using *in vitro* and *in vivo* experimental models of tuberculosis, we demonstrate that a combined activation of cytosolic receptors RIG-I and NOD2 can amplify a protective immune response induced by the BCG vaccine against TB. Infant vaccination using BCG is routine in many countries whereas, in a recent clinical trial, BCG vaccination of elderly showed significant protection against respiratory infections (7). Thus, our data suggests that stimulation of innate signaling pathways through DNA and RNA sensing intracellular receptors is a valid adjuvant strategy for the BCG vaccine.

## Data Availability Statement

The original contributions presented in the study are included in the article/supplementary materials. Further inquiries can be directed to the corresponding authors.

## Ethics Statement

The animal study was reviewed and approved by Institutional Biosafety Committee of University of Texas Health Science center, Houston.

## Author Contributions

CJ designed the study. AK, VKS, AM, AS, SP and SC conducted the experiments and analyzed the data. CJ wrote the manuscript. AK and RPI edited the manuscript. DZ and DHC provided resources and feedback on the manuscript. All authors contributed to the article and approved the submitted version.

## Conflict of Interest

RI is a shareholder of Spring Bank Pharmaceuticals, Inc. AS, SP, and SC own stock options in SBP.

The remaining authors declare that the research was conducted in the absence of any commercial or financial relationships that could be construed as a potential conflict of interest.
